# Urachal endometrioma: a case report

**DOI:** 10.1186/1752-1947-3-9310

**Published:** 2009-12-01

**Authors:** Katherine M Browne, Stephen S Connolly, Niamh Daly, Tom Crotty, Robert J Flynn

**Affiliations:** 1 Department of Urology, Adelaide and Meath Hospitals incorporating the National Children's Hospital, Tallaght, Dublin 24, Ireland; 2 Department of Gynaecology, Adelaide and Meath Hospitals incorporating the National Children's Hospital, Tallaght, Dublin 24, Ireland; 3 Department of Histopathology, Adelaide and Meath Hospitals incorporating the National Children's Hospital, Tallaght, Dublin 24, Ireland

## Abstract

**Introduction:**

We discuss a rare presentation of an unusual case of endometrioma.

**Case presentation:**

A 40-year-old Caucasian woman presented with subacute abdominal pain and a suprapubic mass. A final diagnosis was made after the mass was resected and histopathology confirmed an endometrioma originating from an urachal remnant. Select imaging studies and histopathology are presented in this case report.

**Conclusion:**

While endometriomata are well known to arise from abdominal scars, the condition described in this case report is a rare example of an endometrioma arising from the urachus. A review of the pathological complications of the urachus is also included.

## Introduction

Endometriosis is defined as the presence of endometrial type glands and stroma outside the uterus. The areas usually affected are the fallopian tubes, ovaries, uterine ligaments, ureters and bladder [1]. The term endometrioma is used when endometriosis appears as a circumscribed mass. The most common involvement outside of the pelvis occurs within the lower abdominal wall, caesarean section scars and less commonly the umbilicus. The incidence of endometriosis within an abdominal scar for hysterectomy is estimated at only 1% [2].

## Case presentation

A 40-year-old Caucasian woman presented to the emergency room with a six-month history of progressive lower abdominal pain. She had failed to visit a doctor sooner for fear that she may have a malignancy. Her medical history was notable for hysterectomy and unilateral salpingo-oophorectomy five years prior to presentation to treat endometriosis. Her obstetric history was remarkable for three lower segment caesarean sections, all via a suprapubic (Pfannenstiel) incision. Hormone replacement therapy had been instituted four years previously following the onset of symptoms of oestrogen insufficiency.

**Figure 1:**
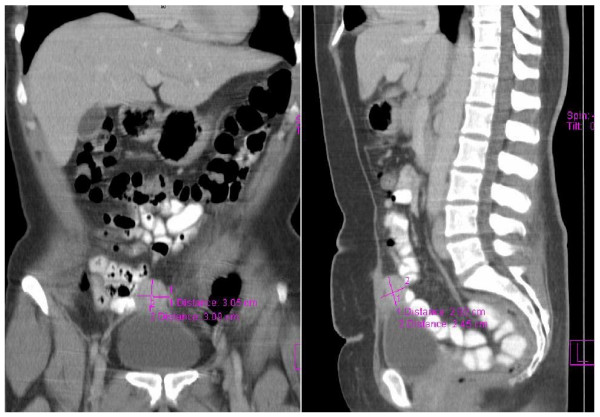
**Selected computed tomography images showing urachal endometrioma**.

A physical examination at the emergency room revealed a 3-cm poorly defined, tender suprapubic mass extending to her umbilicus. The overlying skin was normal and the mass appeared to be tethered to the abdominal wall. No urinary symptoms were present and her urine analysis was clear. Contrast computed tomography of her abdomen and pelvis demonstrated a 3.3-cm lower abdominal mass intimately related to the dome of the bladder in a position that was typical of urachus (Figure 1). Flexible cystoscopy reported the appearance of an extrinsic mass indenting the dome of the bladder, but no mucosal abnormality was found.

A percutaneous trucut (16G) biopsy, which only showed the presence of fibromuscular tissue, proved to be of no help. An open exploration of this urachal mass was performed through a laparotomy incision. No technical problems were experienced intraoperatively. The fibrous mass was distinct and easily separated from the bladder. Wide local excision was performed, but removal of a cuff of bladder was found unnecessary. Histopathological analysis concluded the mass to be a benign endometrioma arising from the urachus. The patient's recovery has been excellent without any recurrence of the pain she previously experienced.

## Discussion

An embryologic structure, the urachus is the canal joining the fetal urinary bladder to the allantois. The urachus, when obliterated normally, forms the median umbilical ligament. Persistent remnants are uncommon but may still manifest clinically as a vesicocutaneous fistula, urachal cyst or umbilical sinus. The presence of urothelium within the persistent urachus has been reported to result in malignant transformation.

A recent article in the American Journal of Surgery retrospectively examined abdominal wall endometriomas and found that the mean age of presentation was at 29.4 years. Presenting symptoms were noted to include abdominal mass, cyclical and non-cyclical pain with dysmenorrhea [3,4]. Although rather uncommon, endometriosis can occur in the postmenopausal (oestrogen-deprived) state [5], and usually occur in women who undergo unopposed oestrogen replacement therapy [6]. Previous case reports have described umbilical endometriosis with periodic bleeding from the umbilicus without prior pelvic or abdominal surgery [7].

As demonstrated in our patient, however, endometriosis may masquerade as a tumour arising from the urachus [8]. Endometriosis can display local aggression, with urinary bladder endometriosis previously reported to extend into the adjacent bowel [9]. Endometriosis of the abdominal wall scars is rare, especially in postmenopausal woman. However, it must be considered as a possible cause of any abdominal wall mass in a woman who has had previous pelvic surgery and who is of reproductive age or taking exogenous hormones. Malignant transformation has been described in abdominal wall endometriosis, with clear cell carcinoma and endometrial carcinoma being the most common reported variants. As such, radical surgery is the most common treatment applied [10].

## Conclusion

This case report illustrates a rare presentation of urachal endometrioma. Accurate final diagnosis can only be accomplished after surgical excision and histopathological examination of the mass. Malignant transformation has been described in abdominal wall endometriosis and radical excision is the mainstay of treatment.

## Consent

Written informed consent was obtained from the patient for publication of this case report and any accompanying images. A copy of the written consent is available for review by the Editor-in-Chief of this journal.

## Competing interests

The authors declare that they have no competing interests.

## Authors' contributions

KB, SC and RF wrote and proofread the manuscript. ND and TC performed pathological work and research. They also contributed in writing the manuscript. All authors read and approved the final manuscript.

## References

[B1] Clement PB Pathology of endometriosis Pathol Annu 1990 25 245 295 2404246

[B2] Chatterjee SK Scar endometriosis: a clinicopathologic study of 17 cases Obstet Gynaecol 1980 56 81 84 7383492

[B3] Bianco RG Parithivel VS Abdominal wall endometriomas Am J Surg 2003 185 6 596 598 10.1016/S0002-9610(03)00072-2 12781893

[B4] Dwivedi AJ Agrawal SN Silva YJ Abdominal wall endometriomas Dig Dis Sci 2002 47 2 456 461 10.1023/A:1013711314870 11855568

[B5] Habuchi T Okagaki T Endometriosis of bladder after menopause J Urol 1991 145 2 361 363 1988732 10.1016/s0022-5347(17)38341-6

[B6] Goodman HM Kredentser D Postmenopausal endometriosis associated with hormonal replacement therapy J Reprod Med 1989 34 3 321 323 2524589

[B7] Zollner U Girschick G Umbilical endometriosis without previous pelvic surgery: a case report Arch Gynecol Obstet 2003 267 4 258 260 12592434 10.1007/s00404-002-0438-9

[B8] Crotty K Endometriosis manifesting as urachal tumour South Med J 1994 87 4 539 540 10.1097/00007611-199404000-00024 8153788

[B9] Stewart WW Ireland GW Vesical endometriosis in a postmenopausal woman: a case report J Urol 1977 118 3 480 481 904066 10.1016/s0022-5347(17)58072-6

[B10] Sergent F Baron M Le Cornec JB Malignant transformation of abdominal wall endometriosis J Gynecol Obstet Biol Reprod (Paris) 2006 35 2 186 190 16575366 10.1016/s0368-2315(06)76394-3

